# Decline in Distribution and Abundance: Urban Hedgehogs under Pressure

**DOI:** 10.3390/ani10091606

**Published:** 2020-09-09

**Authors:** Anouk L. Taucher, Sandra Gloor, Adrian Dietrich, Madeleine Geiger, Daniel Hegglin, Fabio Bontadina

**Affiliations:** 1 SWILD—Urban Ecology & Wildlife Research, Wuhrstrasse 12, 8003 Zurich, Switzerland; sandra.gloor@swild.ch (S.G.); adrian.dietrich@swild.ch (A.D.); madeleine.geiger@swild.ch (M.G.); daniel.hegglin@swild.ch (D.H.); fabio.bontadina@swild.ch (F.B.); 2 Palaeontological Institute and Museum, University of Zurich, Karl-Schmid-Strasse 4, 8006 Zurich, Switzerland; 3 Institute of Parasitology, University of Zurich, Winterthurerstrasse 266a, 8057 Zurich, Switzerland; 4 Swiss Federal Research Institute WSL, Biodiversity and Conservation Biology, Zuercherstrasse 111, 8903 Birmensdorf, Switzerland

**Keywords:** *Erinaceus europaeus*, population trend, population reduction, settlement area, citizen science, urban densification

## Abstract

**Simple Summary:**

Hedgehogs have been found in higher densities in urban compared to rural areas. Recent dramatic declines in rural hedgehog numbers lead us to pose the question: how are hedgehogs faring in urban areas? In this study, we examined how hedgehog numbers have changed in the city of Zurich, Switzerland, in the last 25 years. We compared data collected through citizen science projects conducted in 1992 and 2016–2018, including: observations of hedgehogs, data from footprint tunnels, and capture-mark recapture studies. We found that hedgehog numbers have declined by 41%, from the former average of more than 30 individuals per km^2^, in the last 25 years. In the same time span, hedgehogs have lost 18% of their former urban distribution. The reasons for this decline are still unknown. Intensification of urban buildup, reduction of green space quality, the use of pesticides, parasites, or diseases, as well as increasing numbers of badgers, which are hedgehog predators, in urban areas are discussed as potential causes. Worryingly, these results suggest that hedgehogs are now under increasing pressure not only in rural but also in urban areas, their former refuges.

**Abstract:**

Increasing urbanization and densification are two of the largest global threats to biodiversity. However, certain species thrive in urban spaces. Hedgehogs *Erinaceus europaeus* have been found in higher densities in green areas of settlements as compared to rural spaces. With recent studies pointing to dramatically declining hedgehog numbers in rural areas, we pose the question: how do hedgehogs fare in urban spaces, and do these spaces act as refuges? In this study, recent (2016–2018) and past (1992) hedgehog abundance and distribution were compared across the city of Zurich, Switzerland using citizen science methods, including: footprint tunnels, capture-mark recapture, and incidental sightings. Our analyses revealed consistent negative trends: Overall hedgehog distribution decreased by 17.6% ± 4.7%, whereas abundance declined by 40.6% (mean abundance 32 vs. 19 hedgehogs/km^2^, in past and recent time, respectively), with one study plot even showing a 91% decline in this period (78 vs. 7 hedgehogs/km^2^, respectively). We discuss possible causes of this rapid decline: increased urban densification, reduction of insect biomass, and pesticide use, as well as the role of increasing populations of badgers (a hedgehog predator) and parasites or diseases. Our results suggest that hedgehogs are now under increasing pressure not only in rural but also in urban areas, their former refuges.

## 1. Introduction

Currently, over half of the world’s human population lives in cities and, by 2050, it is estimated that over 66% of people will do so [1]. With this current rise in population, the area covered by urban settlements is expected to triple by 2030 [2]. This massive increase in urbanized land cover has inevitably become one of the greatest concerns of modern conservation [3,4]. In addition, expanding urban spaces are also densifying, with the net result of smaller and more intensively used green spaces [5,6]. Understanding urban ecology is key to conservation efforts in these human-built and dominated landscapes.

Rather than being simply degraded landscapes, urban areas provide habitat for a wide array of wildlife. The urban environment’s particular habitat characteristics render it a unique ecosystem. Although cities tend to be characterized by fewer natural resources, greater anthropogenic disturbance and higher levels of fragmentation than pristine systems, urban areas feature a greater diversity of habitats, more (often anthropogenic) resources, and fewer natural predators [7,8,9,10]. Anthropogenic disturbance creates an urban landscape that is highly variable in temperature, pollution level, habitat availability, and species composition across small spatial scales compared to the non-urban surroundings. These extreme pressures may result in profound behavioral adaptations in the animals inhabiting urban areas [11,12] and, in some cases, may promote rapid evolution [13,14,15,16]. Certain species benefit from the habitat mosaics that are urban spaces [17].

One such species is the European hedgehog *Erinaceus europaeus*, which is commonly associated with the agricultural landscape. This species, however, is also known to inhabit green areas of settlements. It can even reach higher densities in urban spaces than in rural ones [18,19,20,21,22,23]. Factors that provide more favorable conditions for hedgehogs in urban areas include: better habitat quality [19,24], higher anthropogenic food availability [25,26], higher availability of vegetation structures to build day nests [27], and more beneficial climatic conditions [28], coupled with lower risk of predation by badgers [20,29,30]. In multiple European countries, the distribution of hedgehogs has been declining over the past few decades [31,32,33,34,35,36,37]. While hedgehog populations seem to decline over a large range and in several countries, rural hedgehogs are affected particularly strongly by the decline [38]. In the UK, the hedgehog was recently classified as Vulnerable on the Red List [39]. Therefore, the question arises whether urban settlements act as shelters for hedgehog populations.

Although hedgehogs are regularly observed, systematic studies are not easy due to their nocturnal and secretive lifestyles. In addition, access to privately and semi-privately owned land is often limited for researchers. Citizen science is a particularly suitable approach to study urban wildlife because it enables access to privately and semi-privately owned lands, and it allows data collection in a scope otherwise not possible. In addition, the involvement of citizen scientists brings conservation aspects and local wildlife concerns closer to the people who live in cities and offers researchers access to local knowledge. Our recent survey was performed as part of the citizen science project StadtWildTiere (“urban wildlife”) in Zurich, Switzerland, which was established in 2013. This project collects incidental observations of wild animals in various urban areas on an online platform with the aim to increase knowledge on occurrence and distribution, to raise awareness of wild animals in cities, and to promote conservation and mutually beneficial co-existence. Further, the project includes a well-established network of 60 volunteers, who have been trained in various wildlife research methods.

The aim of this study was to test how hedgehogs are doing in urban environments by evaluating the temporal changes in hedgehog distribution and abundance in the city of Zurich in the last 25 years in a case study. We were able to build upon a city-wide call for hedgehog sightings in 1992, combined with a capture-mark-recapture study, which resulted in the creation of a distribution map, as well as an estimate of abundance numbers for a period in the past. In the recent comparative study, we used footprint tunnels [31,40,41], collected hedgehog observations, and employed a capture-mark-recapture approach in four urban districts with the help of citizen scientists to estimate current distribution and abundance measures for urban hedgehogs. The resulting indicators of hedgehog density and distribution were compared over the 25-year period. Such long-term comparative datasets on population development are scarce, and the methodologies inevitably slightly differ between the study periods; thus (with certain caveats in mind), we performed a conservative comparison with the available data. In the wake of the modern biodiversity crisis, it is invaluable to have older data to compare with current estimates.

## 2. Materials and Methods

### 2.1. Study Species and Site

**Figure 1:**
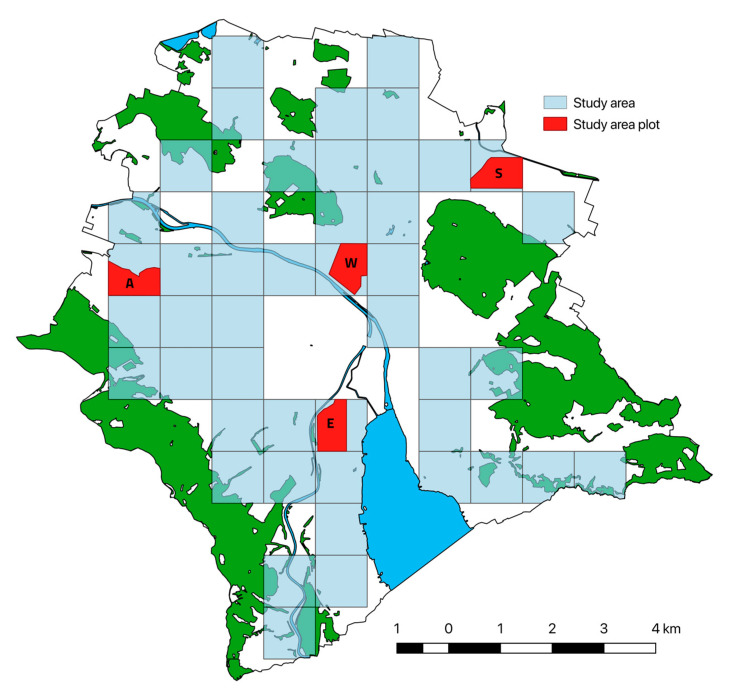
City of Zurich and study areas that were assessed in the course of 25 years (past (1992) vs. recent (2016/2017)). The outline delineates the municipal border of the city of Zurich with forest area (green) and water bodies (blue). Study areas are divided into 1 km^2^ patches (light blue) and study plots (red: Altstetten (A), Enge (E), Schwamendingen (S), Wipkingen (W); in 1992, only Wipkingen was investigated; for details, see text).

We studied temporal changes in the distribution and abundance of urban hedgehogs *Erinaceus europaeus*, in the city of Zurich (433,000 inhabitants, 92 km^2^, 47.369, 8.539), Switzerland. We compared data from two studies, the first of which was carried out in 1992 [42] the second from 2016–2018. Both studies focused on the urbanized area of Zurich, excluding forests and agricultural lands. To allow complete comparability, we focused on a subset of the urbanized area of Zurich, the study area that was assessed in both time periods (46 km^2^, hereafter referred to as the study area), for the abundance estimations (Figure 1).

The data for the abundance estimations were collected in the study area plots, which are sections of the study area (one in 1992 (size 0.23 km^2^) and four in 2016–2018 (size 0.5 km^2^)) and then extrapolated in accordance to the relative return rates of observations or footprint tunnel data to the study area (Figure 1). For the distribution maps, the entire urban space of the city of Zurich was considered (grid of 58 km^2^ representing the urbanized area). To control for observation effort, the observation points from 1992 and 2016–2018 were classed as presence and absence points at a level of ¼ km^2^. This level was chosen to allow for more in-depth comparisons than 1 km^2^ would have done.

### 2.2. Past Study (1992)

In spring 1992, survey cards were sent by mail to approximately 18,000 households (mostly members of conservation and animal protection associations) all over the city of Zurich to ask for observations of hedgehogs. The obtained records of observations were then used to create a distribution map by classing them as presence and absence data. A ¼ km^2^ grid size was chosen. As the cards were not evenly distributed over the city, the proportion of sent out and returned cards per ¼ km^2^ grid cell was used to obtain a relative return rate. Cards were only returned when a hedgehog was seen. It was assumed that all people had the same intention in returning observations after seeing a hedgehog, as they all were members of nature conservation associations.

Intensive hedgehog searches were conducted in one specific plot within the above described study area (hereafter referred to as a study area plot, Wipkingen, 0.23 km^2^) for abundance estimation (Figure 1). The whole area was scanned for hedgehogs once in the course of the night (n = 23), mainly by a single person walking in the dark but occasionally by searching larger areas with a torch. All hedgehogs encountered in this area during a radio telemetry study from beginning of June through end of July 1992 were uniquely marked by attaching six heat-shrink plastic tubes over individual spines with instant glue [19]. This marking lasted at least three months and made possible the unambiguous identification and count of all encountered hedgehog individuals. For estimating abundance, all encountered hedgehogs were summed up. The study area plot was searched intensely, so that it was assumed that the majority of hedgehogs had been encountered. The study area plot was surrounded by either train tracks or heavily trafficked on three sides, and only one hedgehog was observed crossing one of these streets. However, as the study area plot was not truly isolated, it was assumed that some hedgehogs were counted that did not really inhabit that area, while some living in the plot must have been missed. For example, some of the hedgehogs may not have regularly inhabited the study area but only appeared on an occasional exploratory trip or were young, dispersing individuals. To account for this uncertainty, a third of the total amount was used as error range of abundance.

### 2.3. Recent Study (2016–2018)

#### 2.3.1. Surveys of Hedgehog Distribution and Abundance Today

To assess the distribution of hedgehogs, we divided the urbanized area of the city of Zurich into km-squares, 46 of which were surveyed with footprint tunnels made from corrugated plastic (1200 mm × 210 mm × 180 mm) between May and October 2016 [31,40,41]. These 46 km-squares were chosen as they contained the most suitable hedgehog habitat (e.g., no lake and forest) and only areas with the chance of regular sightings by citizens (no peripheral agricultural areas). The estimation of the abundance in both time periods was limited to the study area of the 46 km-squares (Figure 1). Thus, the study area of the early study period (1992) was pruned accordingly.

**Figure A1:**
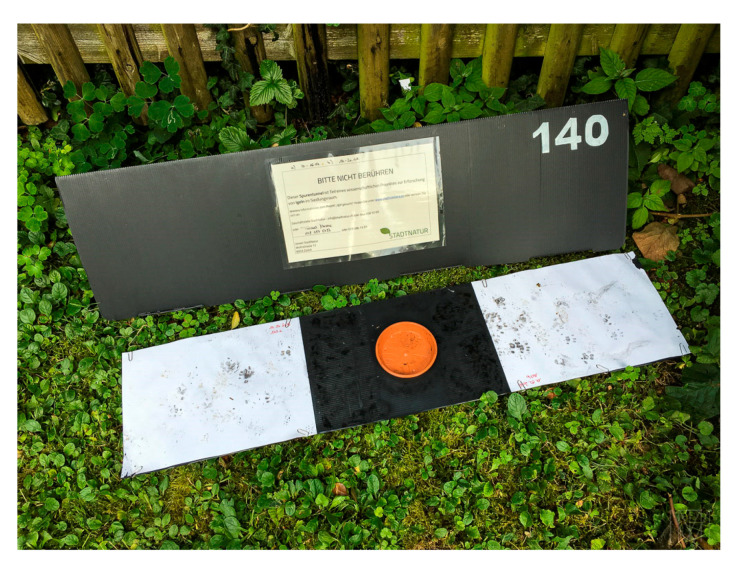
Footprint tunnel with removable plate in front, showing hedgehog footprints on them. Two paper sheets and two brushes of ink are applied to it. A spoon of bait is placed in the brown plate. © Thomas Massie/stadtwildtiere.ch.

Within each study area square (1 km^2^), we defined a 500 m × 500 m square (in cases where no settlement area was included in the square, we chose a 400 m × 600 m rectangle) near the center of that square. We placed ten tunnels within this square (>100 m apart from each other), and checked daily for five consecutive days for footprints and to top up bait and ink. A spoonful of bait (commercially available hedgehog food, Claus—Spezial-Igelfutter, Limburgerhof, Germany), two ink-pads (non-toxic ink: mix of carbon powder and vegetable oil), and two sheets of A4 paper were placed inside the tunnel, on a removable plate (Figure A1, Appendix A). It was expected that, if hedgehogs inhabit the study area, they would encounter these footprint tunnels during their nocturnal forays and enter them to reach the bait [40]. In doing so, their feet would touch the ink and leave species specific footprints on the paper. We placed the footprint tunnels along linear features (e.g., wall, hedgerow, fence, etc.) as these are structures that hedgehogs like to follow when foraging [43]. The proportion of these ten tunnels per km-square containing hedgehog footprints were used for further analyses of hedgehog abundance, 0 (no tunnel with hedgehog footprints) to 10 (all tunnel contain hedgehog footprints), hereafter referred to as hedgehog level; see below. The survey was conducted primarily by volunteers from the StadtWildTiere (“urban wildlife”) project in Zurich, as well as interns, and the authors.

In addition to the footprint tunnels, we collected observations of hedgehogs by the general public on an observation platform, which is part of the citizen science project StadtWildTiere. Further, the project includes a well-established network of volunteers, who have been trained in various wildlife research methods. We asked people to send in observations of hedgehogs through the distribution of leaflets, hangouts, and articles in newspapers. From 2016 through 2018, 4125 observations from mammals, reptiles, and amphibians were collected by 449 users. For the recent study, we analyzed hedgehog observation data from 2016–2018 (n = 1096) to match the total number of observations in 1992 (n = 1011). Due to the differences in data collection between the first and the second study periods, we wanted to account for the possibility that there might be an underlying spatial clustering in the more recent data set resulting from a less systematic distribution of flyers, which may have caused us to underestimate the actual recent distribution. To account for such a bias, we examined the distribution of all observations other than hedgehogs (n = 3029). The mean of non-hedgehog observations per cell was 8.8 (= 3029/343 grid cells, median = 6). We removed all cells from both study periods with fewer than 4 observations of non-hedgehog species (in the recent time period) to ensure a minimum level of observation effort. We then examined distribution differences between the past and the recent study periods using these estimates and found that this method actually resulted in an even larger decline compared to the one described above, with all grid cells included. Therefore, we concluded that potential spatial clustering did not lead to an underestimation of the distribution in the recent study. Consequently, we chose to use our more conservative estimate with all grid cells included for the comparison of time periods.

#### 2.3.2. Capture Mark Recapture Study

In 2017, we carried out a capture-mark-recapture (hereafter CMR) study to obtain estimates of abundance. We selected four study area plots, each 0.5 km^2^ in size (districts Altstetten, Wipkingen, Enge, Schwamendingen, Figure 1). The one area where CMR was conducted in 1992 was 0.23 km^2^ in size and delimited by large roads. In the recent study, the size of the study area plots were 0.5 km^2^ to render them more representable for the area, while still being small enough to be searched within 4 h. These areas were chosen in 2017 for comparisons between study years because they all contained higher than average hedgehog density in 1992 but were found to show exceptionally low (Altstetten and Wipkingen) or high (Enge and Schwamendingen) relative density of hedgehogs, respectively, in the footprint tunnel study of 2016 (see Results). Therefore, we considered these areas appropriate for a comparison between years.

We surveyed each of the four study area plots eight times during 4 weeks in June 2017. Surveys were only conducted in good weather conditions, i.e., if there was no heavy rain, in order to not influence capture rates. While sometimes two areas were surveyed in the same night by different teams, the areas were generally surveyed in subsequent nights. For every survey, a researcher and a volunteer searched for hedgehogs with flashlights between 10 pm and 2 am, scanning the entire study area plot via public paths and green spaces. We captured all encountered hedgehogs and examined them to determine weight, sex, and health status (e.g., presence of injuries or an unusual amount of ectoparasites). We marked hedgehogs uniquely with shrink tubes (see methods from study in 1992). Only adult hedgehogs were marked with shrink tubes, as juveniles could still be easily distinguished by their smaller size at this time of the year. The handling of each hedgehog lasted about 10 min, and the animals were subsequently released on site. The capturing protocols were in accordance with the regulations (1992) or approved by the Veterinary Office of the Canton Zurich through the animal experimentation authorization (ZH079/17).

### 2.4. Analyses

We analyzed the data from the CMR study from 2017 in the program MARK [44] to obtain estimates of hedgehog abundance. As all data was collected within four weeks, we assumed a closed population and used the closed capture model (Huggins’ p and c). All four study area plots (Altstetten, Wipkingen, Enge, Schwamendingen) were analyzed together, nested according to study area plot. To test for overdispersion and the fit of the model to the data, we calculated median c-hat (c-hat; 0.987, sampling standard error = 0.046). Values over 1 indicate overdispersion or a bad fit of the model to the data. Models were ranked by Akaike information criterion (AIC). We averaged the three best models (AIC not more than 3 points apart) to get accurate abundance estimates for the high-density study area plots. The abundance in the two low-density areas was calculated assuming constant survival and recapture probability over time and among study sites. These two different approaches were necessary because the low recapture rates did not allow averaging the models.

**Figure A2:**
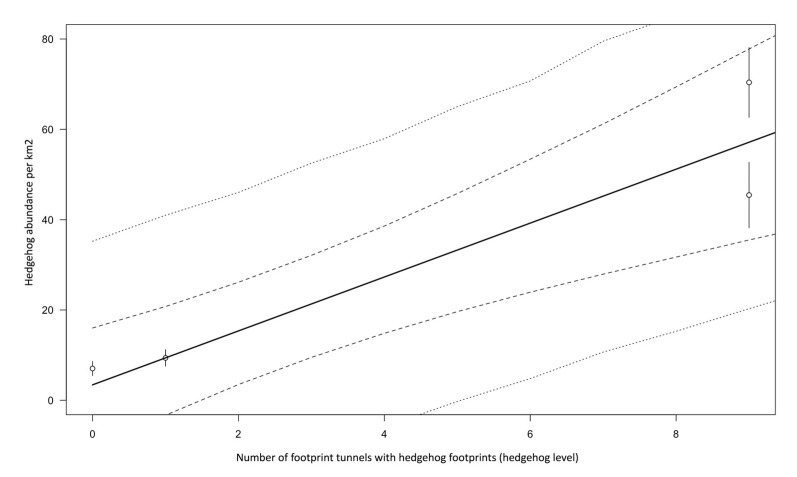
Linear regression between the abundance measures from the capture-mark-recapture (CMR) study (whiskers indicate confidence intervals of the CMR estimates) as the independent variable and the hedgehog levels from the footprint tunnel study (ranging from 1–10) in the respective area as the dependent variable. Regression line in black with confidence (dashed line) and prediction interval (dotted line).

To obtain population size estimates for the study areas where no CMR surveys were conducted (n = 42 km-squares) for the recent study period, we computed a linear regression between the abundance measures from the CMR study as the independent variable and the hedgehog levels from the footprint tunnel study in the respective areas as the dependent variable (Figure A2, Appendix A). Interpolating from the resulting regression equation, we subsequently assigned each hedgehog level across the study area to a corresponding estimate of the abundance. We then summed up all the estimates of the 46 km-squares in the study area, to get an estimate of the population size of hedgehogs in the city of Zurich for the recent study period. The confidence and prediction intervals were estimated using Monte-Carlo simulation in the software Stan [45].

Similarly, we calculated the hedgehog population size for the same study area (46 km-squares) in 1992 using the relative return rate (i.e., the proportion of survey cards returned per ¼ km^2^). For this, we ran a linear regression using the relative return rate as the independent variable and the abundance estimate of the study area Wipkingen (where hedgehog abundance was estimated in 1992 by marking and summing up the encountered hedgehogs) as the dependent variable. Since abundance was only measured in one area, we improved the model by using the y-intercept from the 2016/2017 data (which is 3.4 hedgehogs/km^2^; see results). We considered this a better fit for the model’s intercept than setting it at zero, as even in areas where no hedgehog were detected by the footprint tunnels, there likely is a very low hedgehog density present. This was shown in the two study area plots, Altstetten and Wipkingen, where 1 and 0 out of 10 tunnels contained hedgehog tracks, respectively, yet some hedgehogs were found during the CMR study (4 and 3 individuals, respectively). The hedgehog abundance for each km-square (n = 46) was compared between time periods to get a relative percent change showing areas with relative declines or increases in hedgehog abundance compared to 1992. These estimates were obtained by using the relative return rate of the survey cards (for 1992), and the hedgehog level from the footprint tunnels (for 2016–2018) in combination with the CMR numbers from the respective year. In addition, the km-squares were divided in three abundance categories of low (0–19 hedgehogs per km^2^), medium (20–39), and high (>40) and their frequency in each survey was plotted.

**Figure A3:**
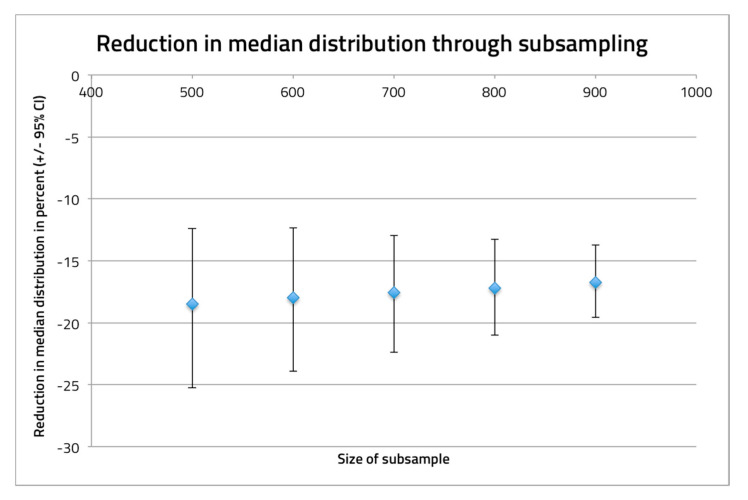
Estimation of the decrease in median distribution area (in percent (± 95% confidence interval), revealed through subsampling. With increasing size of the subsample, the estimate becomes more accurate, on the other side the degrees of freedom become more restricted (because overall sample size = 987 for 1992 and 1096 for 2016–2018). Our estimate resulted by averaging the values for subsample sizes 500 through 900.

We then used the point observation data from 1992 (from survey cards) and 2016—2018 (from the StadtWildTiere platform) to construct hedgehog distribution maps for the two study periods based only on occurrence data (presence/absence of observations) on a ¼ km^2^ level across the entire study area. We chose the level at ¼ km^2^ in order to have enough resolution to compare the data and to have the same sized areas in both time periods. The presence data from the footprint tunnels are included in the data from 2016–2018. We modeled the distribution in both time periods with a subsampling of the data set to estimate the difference in the distribution between both time periods. Our subsampling consisted of randomly selecting 100 observations from the full sample (without resampling, using the sample-function from the r-package “base” [46] and seeing how many ¼ km^2^ grid cells were occupied. We used this stepwise function to produce estimated asymptotic curve that then allowed us to calculate an estimate for the expected distribution (value estimating the horizontal asymptote for large x). We repeated the subsampling and asymptote modeling for subsample sizes of 200 to 900 at increments of 100 and repeated each subsampling 1000 times. With the resulting asymptote estimates (for the subsample sizes 500 to 900), we calculated differences in distribution estimates between the subsamples (Figure A3, Appendix A). This subsampling method was only used to validate the extent of the hedgehog decline in distribution between the two study periods. All analyses were conducted using the programs QGIS (Version 2.18) [47] and R (Version 3.6.2) [48].

## 3. Results

### 3.1. Change of Hedgehog Distribution in 25 Years

**Figure 2:**
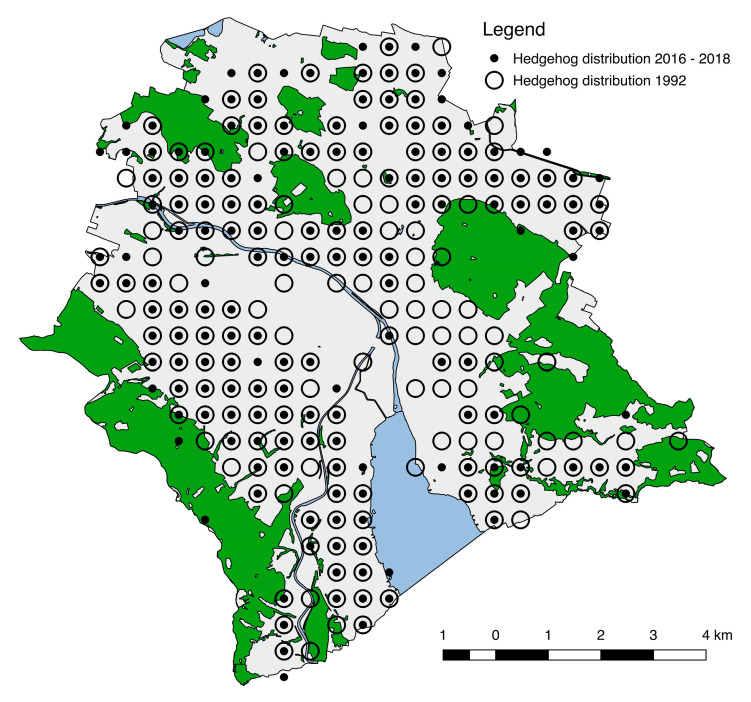
Distribution map of hedgehogs *Erinaceus europaeus* in the city of Zurich, Switzerland in the past study 25 years ago (1992, circles) and in the recent study (2016–2018, points). The outline delineates the municipal border of the city of Zurich with forest area (green) and water bodies (blue). Distribution circles represent the ¼ km^2^ survey grid. The distribution of hedgehogs declined by −17.6% (± 4.7%) in the 25 years between the studies.

In the past study, 1011 observations of hedgehogs were received via returned survey cards (approximately 18,000 where sent in total). Hedgehogs were present in 87.1% of the 232 grid cells (circles in Figure 2). Only parts of the inner-city areas and the industrial areas along the Limmat river, which are densely built and contain little green space, were found to be unoccupied.

In the recent study from 2016–2018, we collected 1096 hedgehog observations on the citizen science platform stadtwildtiere.ch for the entire study area. In the footprint study from 2016, 121 of the 460 footprint tunnels (26.3%) contained hedgehog tracks. Taken together, these surveys indicate hedgehogs currently occupy 74.6% of the 232 grid cells (points in Figure 2).

A comparison of the hedgehog distributions between study periods showed that hedgehogs were found in 30 grid cells (12.9%) in the recent study, which were unoccupied in the past. However, in 25.4% of the 232 grid cells, hedgehogs were missing, despite having occurred there in 1992 (Figure 2). In summary, the presence of hedgehogs reduced by 12.5% of the 232 grid cells in the 25 years between the studies. A subsampling method revealed a reduction in median distribution estimates between the study periods of 17.6% ± 4.7% (mean ± confidence interval, Figure A3, Appendix A).

### 3.2. Change of Hedgehog Abundance and Density in 25 Years

In the past study, 18 individual hedgehogs were marked (June to August 1992) in the Wipkingen study area plot and the density was calculated to be 78.0 ± 26.0 hedgehogs/km^2^ (mean ± confidence interval CI). To calculate population size across the total study area, the relative return rate of the survey cards, the estimate from Wipkingen, and the intercept estimate from the recent study period were used. This allowed us to estimate their abundance as 1477 ± 492 (total ± CI) hedgehogs for the study area in the city of Zurich in 1992.

In the recent study, we caught and marked a total of 57 hedgehogs in four study area plots (30 in Schwamendingen, 19 in Enge, 4 in Altstetten, and 3 in Wipkingen; S, E, A, and W, respectively, in Figure 1; Table A1, Appendix A). All hedgehogs were in relatively good condition. We recaptured individual hedgehogs on average 2.13 times (range: 1 to 6 times, Table A1, Appendix A). Using the estimates from the CMR models, we assigned each hedgehog level across the study area to a corresponding estimate of the abundance and added them to get an estimate for the entire study area. The hedgehog densities in the study area plots were 70.4 (62.6–78.1) hedgehogs/km^2^ in Schwamendingen, 45.4 (38.1–52.7) hedgehogs/km^2^ in Enge, 9.3 (7.5–11.2) hedgehogs/km^2^ in Altstetten, and 7.0 (5.4–8.6) hedgehogs/km^2^ in Wipkingen (estimate (confidence interval), Table A2, Appendix A). Both methods used in the recent study—footprint tunnel and mark-recapture—produced similar results for the different areas of Zurich. Areas with low hedgehog numbers in the footprint tunnels had also low numbers in the CMR study, and vice versa (Figure A2, Appendix A). The population size across the study area in 2017 was 878 (844–910) (total and prediction interval) hedgehogs. A comparison of the total estimated hedgehog abundance for the 46 km-squares studied in both study periods revealed a decline of 40.6% in the 25-year period.

**Figure 3:**
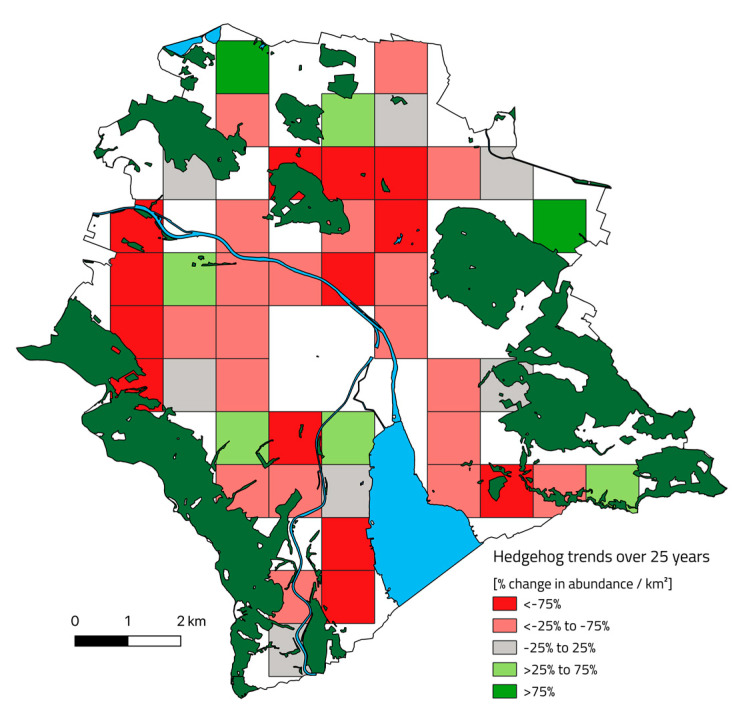
Changes in hedgehog abundance estimates between the past (1992) and recent (2016–2018) study (% change in abundance/km^2^). The outline delineates the municipal border of the city of Zurich with forest area (green) and water bodies (blue). Squares signify the study area divided into km^2^: declining hedgehog abundance (red), increasing hedgehog abundance (green).

**Figure 4:**
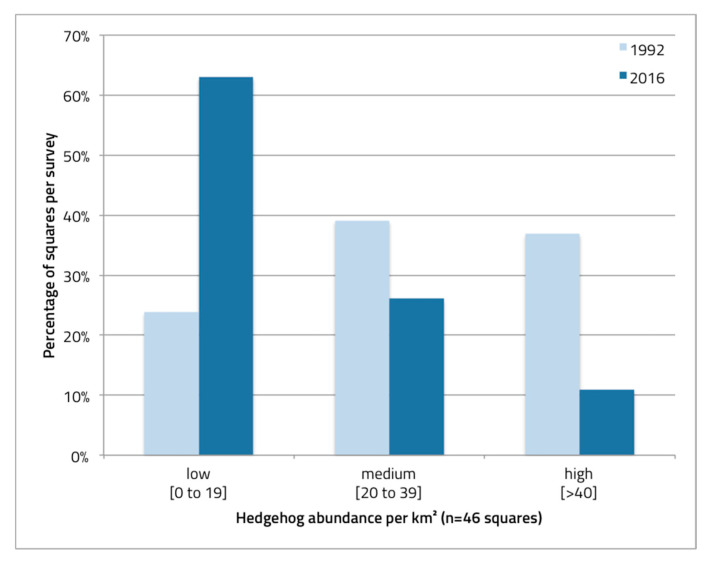
Proportion of km-squares per survey period (past in light blue; recent in dark blue) with low, medium, and high hedgehog abundance per km^2^.

The change in hedgehog abundance per km^2^ varied between study plots and study periods. At the same time, some areas seem to have experienced a more pronounced decline in relative hedgehog abundance than other areas. When plotting the change in abundance for each km^2^ grid, the changes between the recent and past become apparent (Figure 3). Very few areas have seen a relative increase in hedgehog abundance, while most study areas have fewer hedgehogs now than they used to. For the study area plot in Wipkingen, the hedgehog density in 1992 was calculated to be 78.0 ± 26.0 hedgehogs per km^2^, while, in 2017, it was only 7.0 ± 1.6 hedgehogs, which corresponds to a ten-fold decline. In 1992, 76% of squares contained medium to high hedgehog abundances, while, in the recent period, 63% of squares contained low abundances, with only 10% containing high abundances (Figure 4).

## 4. Discussion

Our results suggest that the distribution of hedgehogs in Zurich has reduced over the last 25 years by 17.6%, while hedgehog numbers have declined by 40.6%. This alarming result is in sharp contrast to the expectation that green urban areas provide an ideal habitat refuge for hedgehogs. In 1992, hedgehogs thrived in Swiss cities and occurred in all green areas of the city of Zurich [19,24,42]. The population size in the urban study area of Zurich (46 km^2^) was estimated to be 1477 ± 492 hedgehogs in 1992 or on average 32.1 ± 4.8 hedgehogs per km^2^, with varying hedgehog densities between areas across the city. In the study area plot for Wipkingen, where a CMR study was conducted, the hedgehog density was very high with 78 ± 26 hedgehogs per km^2^.

Today, the situation in Zurich has dramatically changed. Hedgehog abundance has declined to an estimated 878 (844–910) hedgehogs for the same study area in Zurich, or 19.1 ± 5.0 hedgehogs per km^2^. Hedgehogs can still be encountered in many parts of the city, and some areas harbor still high densities of hedgehogs (e.g., the highest density estimate was Schwamendingen: 70.4 hedgehogs per km^2^). This density estimate is still high compared to the recent estimate from the urban areas of Sedan in France (36.5 ± 15.2 hedgehogs per km^2^ [18]) or compared to amenity grassland in England (47.0 ± 9.0 [49]). However, overall abundance, densities and distribution in the city of Zurich are much lower now than they were 25 years ago (Figure 4). In certain study area plots with formerly high hedgehog densities, only very few hedgehogs were found today (e.g., Altstetten and Wipkingen). These data indicate that hedgehog densities have not declined equally across the city in this period. This spatially uneven decline suggests that factors which negatively affect hedgehog populations are not evenly distributed across the city (see below). Alternatively, the temporal onset of factors negatively affecting hedgehogs may be shifted between study area plots. However, this city-wide loss in hedgehog distribution does not seem to follow a pattern easily explainable by the data we currently have at hand.

Hedgehog populations have been declining across different habitats and geographical areas in the last few decades, and different rates of decline in distribution and abundance have been found. Hof and Bright (2016) calculated a hedgehog decline rate of 5.0 to 7.4% in occupied grid cells over a 40-year period in England (1960–1975 and 2000–2015 [32]). Davey and Aebischer (2006) report decline rates of 9.1% (in Scotland) to 37.3% (in Wales) and 30.0% (in England) in abundance over a period of nine years (1995–2004 [50]). Analyzing data sets from multiple studies in the UK, Roos et al. (2012) estimate the average decline in occupancy to be around 40% over ten years [37]. All these estimates come from studies in rural or larger geographical areas. The rate of decline in distribution found in the recent study (which corresponds to a decline of 7.0% over 10 years assuming a linear decline) is higher than the rates described by Hof and Bright (2016) in England, while certainly lower than Roos et al. 2012 rates for the UK. The rate of decline in abundance (which corresponds to a decline of 15% over 10 years assuming a linear decline) is slightly higher than the rates proposed by Davey and Aebischer (2006) for Scotland but lower than the rates of decline for Wales and England. Thus, these numbers from rural areas, when combined with the numbers of decline from our urban study, suggest that hedgehogs are declining in general. On the other hand, a recent study found high juvenile survival rates in a suburban area, pointing towards healthy populations in this habitat [23]. Therefore, our observed population decline might be caused by factors that are patchily distributed and which are not acting on all populations. Nevertheless, our study indicates that even cities, which are suggested to be refuges for hedgehogs, might be in danger of losing that status to some degree.

### 4.1. Potential Factors Negatively Affecting Hedgehog Populations

The reasons for the observed hedgehog decline are currently unknown and could even be multifactorial. Here, we will provide an overview of potential factors negatively affecting hedgehog populations dividing them into six topics: habitat, food, poison, predation, disease, and parasites. This list is not exhaustive; however, we discuss their relative importance, a possible contribution of an extinction debt, and suggest the major avenues of future research.

#### 4.1.1. Habitat

Hedgehog habitat can become uninhabitable through either the loss of the habitat or its deterioration. Habitat may be lost by being rendered inaccessible via fragmentation or by the complete removal of the habitat itself, e.g., by sealing green spaces. Fragmentation can be caused by barriers [51,52], such as roads, train tracks, fences, or walls that cannot be overcome by hedgehogs [31,53,54,55]. The loss of dispersal structures (e.g., removal of hedges [56]) can contribute to the isolating effect of fragmentation on the populations. The major force bringing about such changes in urban areas is densification [50,57]. Densification of urban spaces is a process occurring around the globe to deal with growing urban populations while reducing urban sprawl. During the study period, the city of Zurich’s population grew by 17.1% from 361,000 to 423,000 people, which is accompanied by an intensive densification process resulting in a loss of urban green space in Switzerland [58,59]. This might be one factor that contributed to the decrease of hedgehog populations in Zurich.

Besides the loss of habitat itself, the loss of habitat quality might be threatening hedgehog populations. The intense maintenance of “tidy” gardens and public green (e.g., dense hedges and lack of dead plant material, such as heaps of branches and leaves) leads to the loss of nesting opportunities [38], hiding spots, and shelter for hedgehogs in urban spaces. Additional habitat threats include garden hazards (e.g., pools without exit possibility, uncovered light shafts, electrical fencing, and automatic mowers), where hedgehogs can fall, get stuck, be hurt, or even be killed. Automobile traffic is also a known mortality factor for hedgehog populations, which might be of particular importance to urban and suburban hedgehog populations [54,60]. However, the moderate increase in vehicle numbers and the widespread introduction of zones with reduced driving speed in Zurich do not point towards a recently growing problem [61], but rather a constant risk and a source of background mortality for hedgehogs. Last but not least, changing summer and winter temperatures due to global climate change might limit the hedgehog’s food and water availability in summer and disrupt its hibernating behavior in winter [23,38]. A detailed analysis of habitat characteristics and changes thereof through time are beyond the scope of this paper. However, analyses on temporal changes of the habitat quality in the study area are under way and will help to shed further light on the reasons behind the observed population decline of hedgehogs in the city of Zurich.

#### 4.1.2. Food

The quality of the food and its availability are linked to the quality of the available habitat. Hedgehogs prey on a wide variety of arthropods and mollusks [62]. Therefore, the currently described global decline in insect biomass is likely to have an impact on insectivorous animals, such as the hedgehog [43,63,64].

In the urban habitat mosaic, we expect hedgehog food sources to be patchily distributed. Gardens and parks with local plant species provide valuable habitat elements for hedgehogs. Compost heaps, being humid and warm, contain a variety of insects and were often visited by hedgehogs in 1992. Although no systematic assessment has been conducted, the numbers of compost heaps in Zurich seem to have declined since the study in 1992 (pers. obs.). Since 2013, organic waste has been collected by the city government to produce biogas, and, since then, many people seem to have given up personal compost heaps in their gardens. This depletion of potential food sources might have contributed to the observed decline of hedgehog populations in Zurich and could also explain a spatially patchy pattern. Furthermore, some garden owners might provide artificial food sources for hedgehogs, which has been shown to increase activity levels during the winter when hedgehogs should be hibernating [65]. The importance of supplementary feeding, however, has not been investigated in detail.

#### 4.1.3. Poison

As an insectivorous animal, the hedgehog undoubtedly suffers from the use of pesticides by a reduction in food availability. Further, as opportunistic feeders [62,66], hedgehogs might be exposed to pesticides either directly by ingesting poisoned bait or indirectly by ingesting poisoned prey. Even though the route is largely unknown, studies found residues of anticoagulant rodenticides (warfarin, coumetetralyl, difenacoum, bromadiolone, brodifacoum, flocoumafen) [67] and organochlorine compounds [68] in hedgehogs, even up to 9 months after the use of such in the study area [69]. Hedgehogs are believed to suffer the same exposure and potential effects of anticoagulant rodenticides as other non-target mammals and predatory birds [67]. A study looking at the casualties of the use of banned pesticides in the Canary Islands (Spain) found hedgehogs among the fatalities [70]. However, the illegal use of banned pesticides might just be a fraction of the problem regarding the potential effects of the use of legal pesticides. Previous studies on the effect of pesticides on hedgehogs were inconclusive [71]. Further research is needed to assess the effects of the ingestion (direct or indirect) of pesticides and environmental toxins by hedgehogs. Indirect effects of poisoning or heavy metal accumulation, such as reduced fecundity, reduced lifespan, impaired disease resistance, or poor growth, are likely hard to measure [72,73]. In Switzerland, the amount of herbicides sold decreased over the last ten years, while the amount of fungicide, bactericide, insecticide, akaricide, molluscicide, and growth regulators remained unchanged, even though many pesticides’ effectiveness has increased in the same time period [74]. In Swiss urban areas, we would expect a generally smaller amount of pesticide used compared to rural areas, particularly less use of insecticide and fungicides. On the other hand, a higher rate of rodenticide use, probably the poison most likely to affect hedgehogs, is expected in more densely populated areas due to a higher abundance of rodents, such as rats (but this might not be a global pattern; also see Reference [75]).

#### 4.1.4. Predation

With their spiny defenses, healthy adult hedgehogs are largely safe from most predators, although a few species can occasionally attack hedgehogs. Domestic dogs and cats, both of which might be encountered in higher densities in urban areas compared to rural ones, are known to be able to predate on young or injured hedgehogs [76]. The effect of such potential predation on hedgehog populations needs to be further explored. Predation of hedgehogs by badgers *Meles meles*, however, is often argued as being an important factor controlling hedgehog populations [21,43,77]. Indeed, badgers are not just predators but also food competitors of hedgehogs, and increasing badger numbers, as has been reported in the UK [78] and in Switzerland [61], could potentially cause hedgehog numbers to decline. These issues become even more relevant as badgers in Switzerland were found to increasingly venture into the urban landscape, areas that have so far been considered safe refuge for hedgehogs [20,61,79]. Hedgehogs were found to behave as in a landscape of fear [80]: they avoid areas with high badger densities [22,41,77,79,81,82], keep closer to hiding structures in areas with badgers [20,43], and increase in density after the removal of badgers [83]. On the other hand, there are areas where hedgehogs and badgers are sympatric and are both thriving, whereas there are suitable habitats with neither hedgehogs nor badgers recorded [22]. Therefore, increasing badger numbers and intraguild predation and competition by badgers is unlikely to be the sole explanation for the observed decline of hedgehog populations. However, increasing competition through increasing badger density in combination with a reduction in prey biomass might lead to an intensification of competition between hedgehogs and badgers [43]. In Zurich, badgers have been sighted across almost the entire city, although observations are less common in the city center for both badgers and hedgehogs (data from the StadtWildTiere platform). There are areas with both badger and hedgehog observations, but there are also areas where hedgehog numbers declined in this study without any badger sightings. We therefore conclude that, if badgers are contributing to hedgehog decline, it is not the single factor driving this pattern.

#### 4.1.5. Diseases and Parasites

If diseases are on the rise or a new parasite is spreading this may negatively impact hedgehog populations, especially if animals are already weakened by high stress levels imposed by other factors [84]. With higher population densities in urban areas compared to rural areas [18,19,20,21], diseases and parasites are likely to spread more quickly within urban populations, especially at crowded feeding stations. Furthermore, high levels of parasitism in hedgehogs (e.g., *Ixodes hexagonus, Ixodes ricinus, Crenosoma striatum, Capillaria* spp.) in general [85], as well as documented increases in the abundance of some wildlife parasites relevant to hedgehogs, such as ticks [86,87] and gastropod-transmitted lungworms [88,89], support the idea of parasitism as contributing factor to population declines in this species. A recent study revealed high and fluctuating prevalence rates of *Capillaria aerophila* in the course of the last three decades ranging between 42.8% and 75% in foxes [88]. In the same period, foxes established in high densities in the middle of urban areas [90,91]. Therefore, an increased infection pressure with the infective stage of this parasite, which frequently also infests hedgehogs and can cause weight loss, bronchitis, and pulmonary damage, is likely [92]. At this point, however, no research has yet uncovered such a factor in the hedgehogs’ distribution range, and the regional rescue center for hedgehogs has not recorded any apparent increase in the number or proportion of ill or heavily parasite affected individuals (Annekäthi Frei, pers. comm.). Furthermore, zoonoses originating from hedgehogs merit further research and monitoring, for example, surveying the prevalence of methicillin-resistent *Staphylococcus aureus*, due to its potential to cause severe infections in humans [93,94,95].

#### 4.1.6. Extinction Debt

In addition to all the factors mentioned above, a potential extinction debt might further complicate the topic. An extinction debt describes emerging ecological cost from former habitat destruction [96]. Habitat fragmentation and isolation do not cause the extinction of species immediately, but produce smaller and potentially inbred subpopulations in smaller habitat islands, which may no longer be well adapted to the current conditions and suffer from inbreeding depression [97]. A study examining the spatial genetic structure of hedgehogs found three relative distinct sub-populations in the city of Zurich [52]. If these populations become increasingly isolated due to fragmentation and were to become inbred, they would be less able to adapt to (even small) environmental changes. A study on the extinction rate of urban plants showed that legacies of landscape transformations by agrarian and urban development can last for hundreds of years, and cities might carry a large extinction debt [98]. Therefore, the patterns of decline seen in current hedgehog populations might have been caused decades ago by habitat fragmentation. This could also explain why we see such patchy distribution and density patterns in cities. On the other hand, the relative high reproduction rate of hedgehogs coupled with a high potential in spatial exploration, might help hedgehogs to adjust their distribution to harmful factors more quickly compared to more stationary and slowly reproducing species. In general, it could prove valuable to take evolutionary principles into account when evaluating the causes of extinction [99].

Further research is necessary in order to study which habitat structures in urban areas support healthy hedgehog populations and enable co-occurrence of badgers and hedgehogs. It is crucial to know how hedgehogs navigate the patchy urban food mosaic and what influence individual gardens and supplemental food sources have. Monitoring efforts will have to be implemented to keep track of current and future levels of disease and parasites in urban hedgehog populations.

### 4.2. Comparability of the Studies

There are relatively few systematic studies of wild hedgehogs due to their nocturnal and secretive lifestyle. This is even more the case for systematic surveys on the distribution and density of hedgehogs. Therefore, there is very little data to investigate hedgehog population changes over time, and any such data is extremely valuable, especially regarding the current biodiversity crisis and already reported alarming hedgehog population declines in other parts of Europe. In Zurich, we were lucky to have such a longitudinal dataset. The data in both time periods were not collected with the exact same methodology. In 1992, observations of hedgehogs were elicited through systematic mailings and abundance was estimated through a CMR study in a small study area, delimited by large roads and measuring 0.24 km^2^. In 2016–2018, when a postal questionnaire would no longer have worked, the people of Zurich were asked to send in observations in various ways (through flyers, media releases, and handouts). Additionally, we conducted a systematic study with footprint tunnels in an urban area of 46 km^2^. Furthermore, CMR studies were conducted in four study plots (each 0.5 km^2^). These study plots were purposefully chosen, as they were all areas that had high hedgehog abundances in 1992 (two with still high relative densities and two with low relative densities in the footprint tunnels in 2016). In both time periods, a high but similar motivation of people to report hedgehog sightings was assumed (similar target groups of nature lovers) and, to minimize the potential effect of differing observation effort, observations were analyzed as presence and absence data only. Therefore, we think that potential caveats by slightly different study designs are addressed sufficiently in order to render the different datasets comparable.

The abundance estimates for both years were extrapolated using relative return rates (in 1992) and footprint tunnel levels (in 2016–2018). This allowed us to get more accurate estimates rather than simply extrapolating the estimates from the CMR studies to all study areas. We used three years of data to construct the distribution map in the recent period, so that we could match the amount of observations in 1992 and make sure that with increasing numbers of observations the distribution was not increasing. In fact, the use of multiple years of observations should, if anything, lead to an overestimation of current hedgehog distribution. Even by using these conservative estimates, the pattern of decline in abundance and distribution was clearly confirmed.

## 5. Conclusions

This study is the first to quantify the decline rate of urban hedgehogs in a European city over time. In the light of continental-wide reports of declining hedgehog numbers in rural areas, urban areas have been seen to be the hedgehogs’ refuge from habitat destruction, intensification of agriculture, and, in some places, the recent increase of badger populations. Our results, however, extend the alarming pattern of hedgehogs under pressure to urban areas.

After an evaluation of possible causes of the decline in urban habitat, the major reasons remain unclear. Further research is necessary to investigate the role of habitat deterioration, connectivity, and food supply, as well as the negative effects of predation, diseases and parasites, and pesticide use as potential causes. The patchy pattern of the decline suggests the influence of a single or combination of spatially unequally distributed factors. This evaluation of the causes of the decline is critical, given the alarming decline of this species in the urban area. Based on such further evaluations, conservation measures can be planned and implemented.

Citizen science proved to be a suitable method to investigate urban wildlife and is a promising tool to further investigate the causes of the decline, as well as an aid to implement measures to remedy this loss of urban wildlife. Particularly charismatic animals, such as hedgehogs, are well suited to such work, as they provide the perfect focus to engage a wide public and to raise awareness for conservation.

## Appendix A

**Table A1:** Data sheet of all hedgehog captured during the 2017 capture-mark-recapture (CMR) study.

Study Area Plot	Individual	Sex	Weight (Range in g)	Health Status	Number of Captures
Altstetten	A_A	female	1430	good	1
Altstetten	A_B	unknown	1460	good	1
Altstetten	A_C	unknown	1230	good	1
Altstetten	A_D	female	1000	good	1
Schwamendingen	S_A	female	580–1150	good	3
Schwamendingen	S_B	female	1030–1220	good	4
Schwamendingen	S_C	unknown	860–1010	good	2
Schwamendingen	S_D	unknown	1020	good	1
Schwamendingen	S_E	female	640	good	1
Schwamendingen	S_F	female	710–1120	good	4
Schwamendingen	S_G	female	820–1040	good	5
Schwamendingen	S_H	female	870–950	good	2
Schwamendingen	S_I	female	890–1050	good	3
Schwamendingen	S_J	male	840	good	1
Schwamendingen	S_K	male	790–820	good	2
Schwamendingen	S_L	female	1010–1040	good	2
Schwamendingen	S_M	female	910–980	good	4
Schwamendingen	S_N	unknown	1060	good	1
Schwamendingen	S_O	male	400–830	good	3
Schwamendingen	S_P	female	1130–1260	good	2
Schwamendingen	S_Q	female	1240–1340	good	4
Schwamendingen	S_R	unknown	700–900	good	4
Schwamendingen	S_S	female	1000	good	1
Schwamendingen	S_T	male	1050	good	2
Schwamendingen	S_U	male	920–1080	good	3
Schwamendingen	S_V	male	880–980	good	2
Schwamendingen	S_W	female	1040	good	1
Schwamendingen	S_X	female	470–500	good	2
Schwamendingen	S_Y	male	920	good	1
Schwamendingen	S_Z	female	840	good	1
Schwamendingen	S_AA	female	930	good	1
Schwamendingen	S_AB	female	1210	good	2
Schwamendingen	S_AC	male	1000–1120	good	2
Schwamendingen	S_AD	male	1070	good	1
Enge	E_1	female	1225–1125	good	5
Enge	E_2	male	1275–1375	good	6
Enge	E_3	female	825–875	good	4
Enge	E_4	male	875–1025	good	3
Enge	E_5	female	1225	good	1
Enge	E_6	female	875–925	good	3
Enge	E_7	female	1125	good	1
Enge	E_8	female	1275–1325	good	3
Enge	E_9	female	1275–1325	good	2
Enge	E_10	male	925–1025	good	3
Enge	E_11	unknown	825–875	good	2
Enge	E_12	unknown	975–1075	good	2
Enge	E_13	unknown	675	good	2
Enge	E_14	unknown	1025	good	1
Enge	E_15	female	925–1075	good, medium	2
Enge	E_16	unknown	1125	good	1
Enge	E_17	female	1175	good	1
Enge	E_18	male	1350	many flees	1
Enge	E_19	female	1125	good	1
Wipkingen	W_A	male	1275	good	1
Wipkingen	W_B	female	750	good	2
Wipkingen	W_C	male	1100	good	1

**Table A2:** Population size estimates for the study area plots resulting from the capture-mark-recapture (CMR) data (hedgehogs/½km^2^).

Study Area	Estimate	Standard Error	Unconditional Standard Error
Enge	22.72	3.28	3.63
Schwamendingen	35.19	3.78	3.87
Wipkingen	3.52	0.79	NA
Altstetten	4.69	0.92	NA
